# Influence of grandmothers on exclusive breastfeeding: cross-sectional study

**DOI:** 10.31744/einstein_journal/2018AO4293

**Published:** 2018-10-30

**Authors:** Thelen Daiana Mendonça Ferreira, Luciana Dantas Piccioni, Patricia Helena Breno Queiroz, Eliete Maria Silva, Ianê Nogueira do Vale

**Affiliations:** 1 Universidade Estadual de Campinas, Campinas, SP, Brazil.; 2 Centro Universitário de Indaiatuba, Indaiatuba, SP, Brazil.

**Keywords:** Breast feeding, Lactation, Grandparents, Health knowledge, attitudes, practice in health, Aleitamento materno, Lactação, Avós, Conhecimentos, atitudes e prática em saúde

## Abstract

**Objective:**

To evaluate the influence of grandmothers of infants on exclusive breastfeeding, from their perspective.

**Methods:**

A descriptive cross-sectional study was carried out with 91 women from a hospital in the inland state of São Paulo. By means of a questionnaire, the following data were collected: sociodemographic data of the grandmother, mother and child; duration of exclusive breastfeeding of daughter or daughter-in-law; information on kinship of participant and grandchild; kind of help provided by grandmother; and knowledge about breastfeeding.

**Results:**

The prevalence of infants on exclusive breastfeeding at two months was 35.17%; a total of 67.3% of grandmothers considered it important to give food to babies before six months of life, and 54% considered relevant breastfeeding at regular intervals. Moreover, 40% believed in weak milk and were not aware of signs of sufficient breast milk. Of the grandmothers interviewed, 69% had already offered tea and water to their grandchildren. In the univariate analysis, the maternal grandmother was more involved and close to the pair mother and child.

**Conclusion:**

The presence of grandmothers influences in exclusive breastfeeding.

## RESUMO

**Objetivo:**

Avaliar a influência das avós de bebês sobre aleitamento materno exclusivo, da perspectiva delas.

**Métodos:**

Estudo descritivo transversal, realizado com 91 mulheres de um hospital do interior paulista. Por meio de questionário, foram levantados dados sociodemográficos da avó, da mãe e da criança; o tempo de aleitamento materno exclusivo da filha ou nora; informações sobre grau de contato entre pesquisada e neto; tipo de ajuda que ela forneceu; e conhecimento sobre aleitamento materno.

**Resultados:**

A prevalência de aleitamento materno exclusivo aos 2 meses foi 35,17%; e 67,3% das avós consideraram importante dar comida ao bebê antes de 6 meses de vida. Já 54% acharam importante ter um horário fixo para amamentar. Ainda, 40% acreditavam em leite fraco e desconheciam os sinais de leite materno suficiente. Dentre as avós entrevistadas, 69% já tinham oferecido chá e água aos seus netos. Na análise univariada, a avó materna mostrou-se mais envolvida e próxima da dupla mãe e filho.

**Conclusão:**

A presença da avó influencia no aleitamento materno exclusivo.

## INTRODUCTION

Breastfeeding is the first feeding practice recommended for promotion of child health and appropriate development. According to the World Health Organization (WHO), maternal milk should be the only source of nutrition in the first 6 months of life; after this period, it should supplement other foods offered in an opportune and healthy manner, up until 2 years of age or more. Human milk is the most complete food that exists for the baby until the age of six months. It is easy to digest, it does not overhaul the child’s bowels or kidneys; it is economical for the family budget; it conveys love and caring, and strengthens the bonds between mother and child. On the woman’s side, the act of breastfeeding is also extremely beneficial: it protects the mother from excessive blood loss after delivery by impeding menses; it prevents anemia; and it decreases the chances of the mother developing breast and ovary cancer. It is the single strategy that most prevents childh deaths, besides promoting physical, mental, and psychic health of the child and of the nursing woman.^(^ ^1^ ^)^

Considering several arguments that justify the practice of breastfeeding, it is worth pointing out that children of lower social economical level are more susceptible to health risks, and breast milk protects them from a range of infections. Regardless of the economic class they belong to, maternal milk provides long-term benefits in decreasing the risks of chronic diseases, such as obesity, hypertension, dyslipidemia, *diabetes mellitus* type 1, and allergies. It is estimated that, in the case of diabetes, 30% of occurrences could be prevented if 90% of children up to 3 months did not receive cow’s milk instead of breast milk.^(^ ^2^ ^)^

In order to verify the current situation of breastfeeding in Brazil, the databases of seven national investigations were analyzed. The prevalence of exclusive breastfeeding (EB) in infants younger than 6 months was only 41.0% in the set of surveys. The median duration of EB was 54.1 days (1.8 month), and the median duration of breastfeeding was 341.6 days (11.3 months).^(^ ^3^ ^)^

To change this reality, actions focusing on factors that interfere in breastfeeding are required. Despite the benefits of breastfeeding being well known, it is recognized that breastfeeding is a practice based on subjectivity, influenced by the social context of the nursing mother, as well as by her social network − especially her mother. The act of breastfeeding is permeated with myths, beliefs, and values transmitted by generations; the grandmothers base themselves on their own experiences to interfere positively or negatively, by means of face-to-face, emotional, information and instrumental support.^(^ ^4^ ^)^

A study conducted in 2015 in a unit of the program Family Health Strategy in the municipality of Cárceres (MT), aimed to learn about the experience of mothers regarding breastfeeding and events that led to early weaning. It was noted that grandmothers can influence negatively in maintaining breastfeeding, especially when it is exclusive. They carry with them a cultural heritage, supported by the empiric knowledge of their ancestors, and with their wisdom based on common sense, they seek to pass on these teachings when they offer tea to the baby, or when they affirm that breast milk is weak/insufficient. Nevertheless, these actions go against the more appropriate scientific proofs that the mother should adopt as to breastfeeding and her child’s care.^(^ ^5^ ^)^

On the other hand, an investigation carried out in Palmeira das Missões (RS) demonstrated the relevance of the role of grandmothers in encouraging breastfeeding. One of the main results of this study was that, within the context analyzed, the influence of the grandmothers proved to be a determining factor for the continuity of breastfeeding or for early weaning. Grandmothers are important as to the transmission of knowledge, wisdom, and experiences related to infant feeding. The nursing team could support autonomy of the subjects, considering co-responsibility and cultural congruence, as well as the interactions of the family group, who – in its dialetic action - modulates, and is modulated by the culture. In the environment analyzed, the study allows inferring that the orientation given to daughters and/or daughters-in-law about infant feeding, is part of the social role of the grandmother, whose experience is recognized as an important cultural value. Additionally, breastfeeding is valued by the grandmothers.^(^ ^6^ ^)^

In Itabuna (BA), a study was performed with a qualitative approach to capture the social representations about breastfeeding of women of the same family from three distinct generations, and to identify continuity and discontinuity in the process of breastfeeding. For women of these three generations, the importance of their mothers’ and/or grandmothers’ experiences was a defining force in the formation of the meanings and conducts that permeate the practice of breastfeeding their children. The women acknowledged the importance of support and encouragement of the family, especially of their mother, for breastfeeding.^(^ ^7^ ^)^

The negative influence of the grandmother’s advice about breastfeeding seems to be related to incorrect knowledge and not to intentionality. When they have adequate information, the role of the grandmothers regarding their grandchild’s feeding is susceptible and modifiable, resulting in better patterns of feeding. During the puerperium, grandmothers can convey to their daughters/daughters-in-law much useful information; some that can hinder breastfeeding as well.^(^ ^8^ ^)^

A study carried out in Australia had the objective of evaluating if the attitudes relative to infant feeding were associated with duration of breastfeeding. It was noted, among other results, that the attitude of the grandmothers about the importance and the practice of breastfeeding influenced the type of infant feeding, as well as duration of breastfeeding within a 12-month period. Mothers who perceived that their own mothers were ambivalent regarding breastfeeding, or who preferred formula feeding were 96% more likely to interrupt breastfeeding at 26 weeks, and had more than double the probability of quitting breastfeeding within 52 weeks.^(^ ^9^ ^)^

A recent systematic review including cross-sectional investigations, cohort, prospective studies, and one randomized controlled study, aimed to quantify the impact of the grandmother in influencing breastfeeding practices of mothers. The eligible studies reported the duration of exclusive breastfeeding and included estimates of the effect of the influence of a grandmother, including or not that person who lived with the baby’s family, the grandmother’s education level, and the attitudes relative to the experience of breastfeeding. This review found evidence that grandmothers have the ability to influence exclusive breastfeeding, and that programs that assist the pregnant and puerperal women should include grandmothers in their interventions, in order to reach maximal impact.^(^ ^10^ ^)^

Grandmothers can negatively or positively influence in duration of breastfeeding. Their presence is a determining factor for the continuity or not of breastfeeding, as they express judgments, and cause varied responses in the nursing mother. Moreover, the support given by the grandmother, when present, is clearly perceived as a facilitating element for continuity of breastfeeding up to 6 months, especially when there is transfer of prior learning to the daughter.

## OBJECTIVE

To assess the influence of grandmothers in exclusive breastfeeding of their grandchildren from their own perspective.

## METHODS

This is a descriptive cross-sectional study with 91 grandmothers who came for a clinical visit to the menopause outpatient clinic, at a teaching hospital in the interior of the State of São Paulo. Their grandchildren should be aged 6 months to 2 years, and the contact with daughters or daughters-in-law should be daily or weekly. The sample calculation took into consideration the prevalence of EB of 39.1%, and the estimate of probability of EB in children of the State of São Paulo was 10%.^(^ ^2^ ^)^ Data were collected from August to December 2011.

All participants were informed and signed an Informed Consent, as per the rules proposed by the Declaration of Helsinki and its modifications (Declaration of Helsinki, 2000), as well as Resolution 466, of December 12, 2012, by the National Health Council.

The data were collected by an instrument prepared specifically for this investigation, according to the objectives proposed, and organized as frequency tables. To verify possible associations between exclusive breastfeeding and the influence of grandmothers, the χ^2^ test and the Fisher’s exact test were used. The significance level adopted was 5%, that is, p<0.05.

## RESULTS

As to time of EB, 64.83% of the babies were breastfeeding exclusively for 2 months, at the most.

A total of 91 grandmothers participated in the survey. Most mothers were aged between 20 and 29 years and had more than 9 years of education, while 72.53% of the grandmothers had less than 9 years of education. There was a balanced distribution between paternal grandmother and maternal grandmother; most of them had daily contact with the daughter/daughter-in-law and the grandchild after birth.

Knowledge of the grandmothers on breastfeeding proved deficient in various aspects investigated. Most (60%), however, did not believe in the existence of weak milk ( Table 1 ).

**Table 1:** Knowledge of grandmothers on breastfeeding

	n (%)
How long is breastfeeding important	
2 years or more	26 (28.57)
1 year	35 (38.46)
1 year or less	23 (25.27)
Until when one wants	7 (7.69)
Age to start giving food to babies, months	
<6	61 (67.03)
≥6	30 (32.97)
Believes that breast milk can be weak or little	
Yes	36 (39.56)
No	55 (60.44)
Frequency at which babies need to be breastfed	
On demand	42 (46.15)
Set hours	49 (53.85)
Signs that the mother has enough milk (weight gain)	
Yes	6 (6.59)
No	85 (93.41)
Signs that the mother has enough milk (frequency of voiding)	
Yes	1 (1.10)
No	90 (98.9)

When questioned as to the manner in which they helped the mothers, most were involved with household chores and the baby’s hygiene, but declared that they had not participated with any type of help in breastfeeding. Most of them recognize that in some way they had had a positive influence on breastfeeding, even if in some cases, with no success ( Table 2 ).

**Table 2:** Modalities of help in activities carried out by the grandmothers in care of mothers and babies

Modalities of help	n (%)
Bathing the baby	
Yes	61 (67.03)
No	30 (32.97)
Changing the umbilical dressing	
Yes	50 (54.95)
No	41 (45.05)
Changing diapers	
Yes	71 (78.02)
No	20 (21.98)
Providing tea or water	
Yes	62 (68.13)
No	29 (31.87)
Help with the house	
Yes	56 (61.54)
No	35 (38.46)
Help with laundry	
Yes	56 (61.54)
No	35 (38.46)
Help with the kitchen	
Yes	53 (58.24)
No	38 (41.76)
Help receiving visits	
Yes	46 (50.55)
No	45 (49.45)
Help with breastfeeding	
Yes	6 (6.59)
No	85 (93.41)
Influence in duration of breastfeeding	
Encouraged	43 (47.25)
Encouraged, but was not successful	15 (16.49)
Did not influence	32 (35.16)
No information	1 (1.10)

A univariate analysis was carried out with the variables studied and the kinship of the grandmothers (maternal or paternal), revealing some significant and important aspects for understanding about whether there was any difference in the performance of the grandmothers related to their being mothers or mothers-in-law of the puerperal women. The maternal grandmother was closer to the nursing mother and the baby, involving herself more directly in the care of the baby and the home ( Table 3 ).

**Table 3:** Univariate analysis of the variables related to activities of grandmothers, as per kinship (maternal or paternal grandmother)

Variable	Parentesco		p value

	Maternal grandmother n (%)	Paternal grandmother n (%)	
Contact of grandmother and mother			0.03*
Daily	42 (82.35)	25 (62.50)	
Weekly	9 (17.65)	15 (37.50)	
Bathing the baby			0.03*
Yes	39 (76.47)	22 (55.00)	
No	12 (23.53)	18 (45.00)	
Changing the umbilical dressing			0.03*
Yes	33 (64.71)	17 (42.50)	
No	18 (35.29)	23 (57.50)	
Changing diapers			0.03*
Yes	44 (86.27)	27 (67.50)	
No	7 (13.73)	13 (32.50)	
Help with the house			0.11
Yes	35 (68.63)	21 (52.50)	
No	16 (31.37)	19 (47.50)	
Help with laundry			0.11
Yes	35 (68.63)	21 (52.50)	
No	16 (31.37)	19 (47.50)	
Help with the kitchen			0.02*
Yes	35 (68.63)	18 (45.00)	
No	16 (31.37)	22 (55.00)	
Help receiving visits			0.07
Yes	30 (58.82)	16 (40.00)	
No	21 (41.18)	24 (60.00)	

p value <0.05.

## DISCUSSION

The data collected suggests that grandmothers, especially maternal, occupy a prominent position in the family that experiences the birth of a baby, since in most cases, they reported having contact with their daughters and grandchildren.

By crossing the variables with the kinship of grandmothers (maternal or paternal) it was noted that the maternal grandmothers, besides being present more often, had a range of possibilities to help in the daily home life and in care for the grandchildren. A study carried out in the city of Recife (PE) with 158 infants, showed that among the members of the family who influenced breastfeeding, the maternal grandmothers had an important position. The practice exerted by the maternal grandmother in home activities and in care of the newborn, showed an association with the time of EB.^(^ ^11^ ^)^

On the other hand, there are studies showing that the proximity of the grandmother − especially the maternal grandmother − has a negative impact on EB. A study conducted in Porto Alegre (RS) demonstrated an increased prevalence of breastfeeding in the first year of life, especially when the mothers did not live with their own mothers, that is, with their child’s maternal grandmother.^(^ ^12^ ^)^ This result is similar to that of a research carried out in the United Kingdom where, in general, the mothers with frequent contact with their mothers and mothers-in-law were associated with lower rates of breastfeeding initiation and a shorter duration of it. However, cohabitation of the mother with her mother-in-law did not fit into this tendency, that is, daily living of the mother with paternal grandmothers was associated with fewer probabilities of early weaning.^(^ ^13^ ^)^

Most of the grandmothers in this study considered that: one year is the important and fundamental period for breastfeeding; the introduction of solid foods should begin before the sixth month of life; and the baby needs well-established schedules for breastfeeding, which is contrary to feeding on demand. Breastfeeding rates are discrepant and depend on social and demographic factors. Nevertheless, the WHO, endorsed by the Ministry of Health, recommends breastfeeding for 2 years or more, and exclusively during the first 6 months.^(^ ^2^ ^)^

This study showed that 69% of grandmothers offered water or tea to the infants, which leads one to believe in the relation with beliefs and myths that exist in the population, the object of a study conducted in Viçosa (MG), in which myth is defined as the “representation of real facts or characters, exaggerated by popular imagination, by tradition.” This study showed that one of the main myths is that breast milk does not quench the baby’s thirst, highlighting the importance of offering water and/or tea to the baby in the first days of life, with the purpose of calming the child, relieving ear aches, preventing and treating colds, and especially, quenching the child’s thirst. Indeed, the grandmothers are rarely against breastfeeding; on the other hand, they significantly influence as to the introduction of water or daily infusions to the baby, involuntarily interfering in the success of breastfeeding.^(^ ^14^ ^)^ Another interesting aspect related to offering liquids to babies, is the fact that it is possible that the concept of exclusive breastfeeding is not clear to women, since they understand practicing it would mean to not give any other type of milk, but that they can give other fluids.^(^ ^15^ ^)^ As to the fact of the mother choosing and maintaining EB, the opinions of the father of the child and of the maternal grandmother are relevant. A study carried out by the Food and Drug Administration showed that the probability of mothers breastfeeding exclusively in the first weeks after birth is higher among those who perceive that the father or maternal grandmother of the baby support EB, without the use of water, teas, or supplementation with formulas.^(^ ^16^ ^)^

Another aspect to consider is the fact that most of the grandmothers (53.85%) considered it important to have rigid schedules for breastfeeding, which again demonstrates their lack of knowledge, since what is recommended is to encourage breastfeeding on demand, with no restrictions of time and duration.

It is interesting to note that 40% of grandmothers affirmed that they believed that maternal milk could be weak or insufficient, but they did not know how to objectively identify if the baby is duly fed or not. Weak breast milk is a belief that exists and that can be passed on from generation to generation. A cross-sectional study performed in São Paulo analyzed the primary causes of early supplementation by mothers; 17.8% of them answered they supplemented because their milk “was weak”, or “did not sustain” the baby. It is important to point out that “…there is no weak milk. Every woman, even if she is malnourished (as long as it is not severe malnourishment), produces milk of a good nutritional quality that satisfies all needs of the baby during the first six months of life”.^(^ ^7^ ^,^ ^17^ ^)^

Several authors identified the grandmothers as role models to be followed by the mothers. They also help in housekeeping activities, indirectly supporting lactation. However, sometimes grandmothers discourage natural breastfeeding because of their own experiences. This reveals that, to support breastfeeding, it is necessary to go beyond knowledge about its benefits or handling techniques, and seek the intent of the woman in the act of breastfeeding, taking into account that the nursing mother is more vulnerable to advice and pressures of third parties.^(^ ^17^ ^,^ ^18^ ^)^

Considering the proximity of the grandmothers and their help in caring for the mothers and babies (bathing, changing the umbilical dressing, diaper changing, helping with the house, laundry, and the kitchen), one can infer that the grandmother has credibility and great potential to exert positive influence in the establishment and maintenance of breastfeeding, as long as they acquire knowledge and adequate skills. Their influence is considered positive when mothers or mothers-in-law have accumulated experiences and the important significance of breastfeeding.^(^ ^19^ ^)^

As to making decisions on breastfeeding, often the mothers see themselves in the difficult situation of having to choose between the affirmations of health authorities and the traditions of grandmothers. In this case, grandmothers and family members participate as a relevant source of information about breastfeeding, whose negative/positive influence could justify the incidence and prevalence rates of breastfeeding we observe today.^(^ ^17^ ^)^

One can consider that the grandmothers, especially the maternal grandmothers, can equally support or discourage breastfeeding. One cannot say, however, that they are the only influence, since a myriad of factors, from social and cultural to economic factors are at stake. The older generation, particularly the grandmothers of the baby, play a central role in various aspects of decision making regarding pregnancy and raising children within the family unit. This is especially true in countries with low and medium income rates.^(^ ^10^ ^)^ The opinion of the grandmother is valued since she is the heiress of her own knowledge, coming from her experience acquired over many years, which makes her recognized and respected by the members of her primary group. Isto confere segurança à jovem mãe quando alimenta seu filho.^(^ ^18^ ^)^

The bibliographic review that discussed the interface between family and breastfeeding, emphasizing the importance of the experience of the older generations in the process of teaching and learning to the new generations, showed that the transmission of knowledge between generations sustains values, standards, and beliefs that assure cultural continuity, and it is fundamental that mothers, daughters, and grandmothers interact in this relationship, which can be affectionate or troubled, within the familial space.^(^ ^20^ ^)^

With this perspective, the grandmothers in this study proved to be present and involved. Although they did not recognize the attribution of any value or significance in the breastfeeding process of their daughters/daughters-in-law, they participated in a dynamic familial ritual that spans generations with its unique symbolism. Breastfeeding is a social act permeated by relationships of affection or conflict, which has generated many studies in which biological and behavioral aspects predominate.^(^ ^20^ ^)^

A descriptive and qualitative study carried out in Londrina (PR), aimed to understand the reports of mothers of support received from the healthcare service, and the determining factors in their choice of feeding their children for the first 6 months of life, analyzing vulnerability points for not nursing their child. This research project concluded that mothers feel the need to be supported since the prenatal care to the puerperium, both by the family and healthcare professionals, focused on the difficulties experienced throughout the process of breastfeeding. Within the family environment, regardless of the relationships being affective or conflicting, they stimulate behaviors and subjectivity of women during the cycle of generations. These experiences are cumulative and of great social and cultural value.^(^ ^21^ ^)^

Within the realm analyzed, this study allowed inferring that the orientation to daughters and/or daughters-in-law about feeding of infants, is part of the social role of the grandmother, whose experience has an important cultural value; in addition, breastfeeding is valued by grandmothers.

There is a need to seek new manners of seeing and caring for the family in day-to-day living, especially the family that is experiencing the process of breastfeeding. It is necessary to consider intergenerational knowledge and the social support of the family, so that the woman-mother-nursing mother might breastfeed in a calm manner and be able to care for the new being who arrived in the world, embedded in the knowledge acquired in the family group she belongs to, in that of their mothers-in-law, as well as in the other care systems. For this, professional development of the healthcare team with a family-centered approach, seeking interdisciplinary knowledge on social, human, and biological sciences, in such a way that makes it possible to approach the family more closely, in its parts and as a whole.

The limitations in this study are related to planning the research, which did not include open-ended questions that would have allowed a better exploration of opinions of the grandmothers relative to their contact with their daughters, daughters-in-law, and grandchildren.

## CONCLUSION

The grandmothers did not have a clear idea as to the influence of their proximity with the mother-baby dyad relative to the practice of breastfeeding their grandchildren. Most of the children have a time of exclusive breastfeeding much shorter than what is recommended. The knowledge of the grandmothers was deficient. However, most recognize the inexistence of “weak milk.” The grandmothers (especially the maternal) get much more involved in domestic activities than directly with the mother and child. In the univariate analysis, the maternal grandmother proved to be much more involved and close to the mother and child pair.

Breastfeeding is a learned practice, transmitted from mothers to daughters and influenced by cultural factors, which are experiences resulting from empirical knowledge accumulated throughout life and habitually passed on during the puerperium.

Breastfeeding proved to be strongly linked with the social and cultural context of the nursing mother and her social network; both served as support or generators of conflict, influencing the attitudes of mothers regarding lactation.

The study revealed the importance of including family members - in this case, the grandmothers – within the context of care for the infant’s mother, since in this cultural environment, there seems to be a valuation of the bonds established between these women, which favors the intergenerational transmission of knowledge, experiences, and insights.
